# Impact of NH_4_OH treatment on the ion exchange and pore characteristics of a metakaolin-based geopolymer†

**DOI:** 10.1039/d4ra03972f

**Published:** 2024-06-20

**Authors:** Jing Li, Sarah Mailhiot, Mohammad I. M. Alzeer, Tero Luukkonen, Anu M. Kantola, Ville-Veikko Telkki, Paivo Kinnunen

**Affiliations:** a Fibre and Particle Engineering Research Unit, Faculty of Technology, University of Oulu P. O. Box 4300 FIN-90014 Oulu Finland jing2.li@cea.fr; b NMR Research Unit, Faculty of Science, University of Oulu P. O. Box 3000 FIN-90014 Oulu Finland; c NIMBE, CEA, CNRS, Université de Paris Saclay, CEA Saclay 91191 Gif-sur-Yvette France

## Abstract

We investigated the viability and influence of NH_4_OH post-synthetic treatment on the pore characteristics of geopolymers. Geopolymers are a class of materials with amorphous aluminosilicate three-dimensional frameworks, regarded as amorphous analogues of zeolites. Similar to zeolites, when geopolymers are used in catalysis or adsorption applications, post-synthetic treatments such as ion exchange with NH_4_^+^ salts (*e.g.*, NH_4_Cl and NH_4_NO_3_) and desilication (using strong bases such as NaOH) are necessary to introduce active sites and modify their pore structure, respectively. Recently, it has been shown that treatment with NH_4_OH combines these two steps, in which acidic sites are introduced and the pore structures of zeolites are modified simultaneously. Considering the increasing interest in geopolymers in catalysis and adsorption applications, understanding the impact of such treatment on the structure of geopolymers is needed. Our diffuse reflectance infrared Fourier-transform spectra show that NH_4_^+^ exchanges Na^+^ in the geopolymer, and laser diffraction with scanning electron microscopy images show that the particle size of the powdered geopolymer decreases after NH_4_OH treatment. N_2_ sorption isotherms and ^129^Xe and ^1^H NMR measurements revealed information about the changes in pore structures: micropores were larger than mesopores and inborn mesopores increased in diameter, thereby reducing the surface area to volume ratio. However, pore accessibility and pore connectivity were not altered by NH_4_OH treatment. Since solid-state NMR and X-ray fluorescence revealed desilication, these changes in particle size and pore characteristics are considered to be due to desilication caused by NH_4_OH treatment.

## Abstract 2

The viability and influence of NH_4_OH post-synthetic treatment on a metakaolin-based geopolymer were investigated.

## Graphical abstract

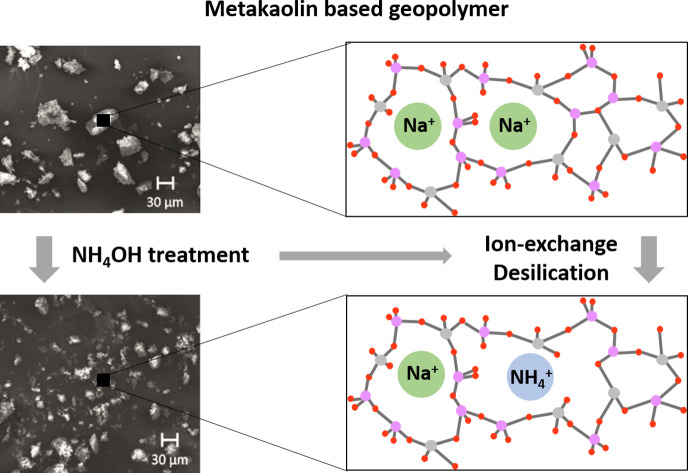

## 1. Introduction

Geopolymers are a class of amorphous aluminosilicate inorganic polymers synthesized through the alkali-activation of aluminosilicate precursors (*e.g.*, natural clays or low-Ca slags).^1^ They could be described as the amorphous analogues of zeolites because of their disordered three-dimensional frameworks.^2^ Compared with synthetic zeolites, geopolymers are cheaper and more easily synthesized at low temperatures (<100 °C).^3^ In our recent studies, we showed that the pore structures of geopolymers include inborn and interconnected micropores and mesopores.^4,5^ Geopolymers have shown good performance as catalysts and adsorbents in some processes, such as NO_*x*_ reduction,^6^ oxidation of volatile organic compounds (VOCs)^7^ and water purification.^8,9^

For catalysis applications, acidic sites within a geopolymer framework have been generated *via* ion exchange of charge-balancing alkali cations (*i.e.*, Na^+^ or K^+^) with NH_4_^+^, followed by thermal treatment, which releases gaseous NH_3_ and forms a H^+^-geopolymer.^10^ This is typically performed by treating a pre-formed geopolymer with a solution of ammonium salt (*e.g.*, NH_4_Cl and NH_4_NO_3_). In addition, ion-exchange procedures have also been used to incorporate catalytically reactive metals or metal oxides (*e.g.*, Ni, Fe, Ce, Co, Cu, and Pt) within a geopolymer framework.^2^ Such cations have been shown to be more efficiently incorporated into an NH_4_^+^ containing geopolymer compared to an originally synthesised Na^+^/K^+^-geopolymer.^2,11,12^ In such applications, the pore characteristics (*i.e.*, surface area and pore volume) of geopolymer catalysts are essential. This has been shown to be significantly improved by applying post-synthetic treatments such as dealumination (treatment with a weak acid) and desilication (treatment with a strong base).^13^ Such post-synthetic treatments are routinely carried out on zeolite catalysts, in which they generate additional interconnected and accessible mesopores.^14^ Similar procedures have been shown to be applicable to geopolymer catalysts.^13^ The effect of such treatments on the geopolymer properties has been studied to some extent by diffuse reflectance infrared Fourier-transform (DRIFT) spectroscopy for monitoring the success of the NH_4_^+^ ion-exchange,^15^ and ^27^Al and ^29^Si magic angle spinning nuclear magnetic resonance (MAS NMR),^16^ which provide information about structural changes caused by the treatment. However, more information about the impact of such treatments on modifying the chemical structure, particle morphology, and pore properties, importantly pore-connectivity, is still needed.

When probing the pore structures of the geopolymers, N_2_ sorption is a widely used method to provide information about mesopores.^17^ Previously, ^129^Xe and ^1^H NMR have been shown to be efficient methods to investigate the pore structures of geopolymers^4,5^ as well as zeolites, silica gels and cementitious materials.^18–21^ By introducing Xe and H_2_O as probe fluids into pores, three of the most relevant porous characteristics, pore accessibility, pore connectivity and pore sizes, can be determined. ^129^Xe NMR spectra provide information about the pore size and accessibility, as ^129^Xe chemical shift (*δ*) is inversely proportional to the pore size^22^ and variable temperature ^129^Xe spectra enable the determination of the mesopore sizes.^23^^129^Xe spin–spin relaxation time (*T*_2_), spin–lattice relaxation time (*T*_1_) and exchange rates of Xe gas between different pores (*k*)^24^ reflect the pore connectivity.^5 1^H *T*_1_ and *T*_2_ relaxation times of H_2_O also reveal information about the pore structure, such as pore size, pore connectivity, and information about the pore surface characteristics.^4,19^ Two-dimensional (2D) relaxation experiments, such as *T*_2_–*T*_2_ (ref. ^25^) and *T*_1_–*T*_2_ (ref. ^26^ and ^27^) experiments, provide higher resolution and more detailed exchange information than 1D measurements.^4^ Pore size distributions can be measured by 1H NMR cryoporometry,^28,29^ which relies on the fact that the melting point of water in a small pore is inversely proportional to the pore size. The amount of unfrozen water is detected as a function of temperature by ^1^H spin echo or Carr–Purcell–Meiboom–Gill (CPMG) experiments. The derivative of the signal intensity is converted to pore size distributions using the Gibbs–Thompson equation.^30,31^

In this work, we report, for the first time, the impact of mild NH_4_OH treatment on the chemical and physical properties of geopolymers. We investigate the dual role of NH_4_OH; the ion exchange of Na^+^ with NH_4_^+^ simultaneously upon modifying the pore structure of geopolymer by desilication (due to the basicity of NH_4_OH). The impact of NH_4_OH treatment on zeolite catalysts has been reported,^32^ but as far as we are aware it has not been reported on geopolymers previously. Modified geopolymer catalysts with post-synthetic treatments have been previously produced *via* multistep synthesis procedures involving an initial NH_4_^+^ ion-exchange step, followed by dealumination, desilication and then an additional NH_4_^+^ ion-exchange step. NH_4_OH produces desilicated geopolymer in its NH_4_^+^ form in a one-pot process without the need for an additional ion exchange step. Herein, the NH_4_OH (0.02 M) treatment was performed on a metakaolin-based geopolymer, previously treated with acetic acid (0.1 M). The chemical structure and particle morphology of the modified geopolymer were thoroughly investigated at different NH_4_OH treatment durations. Special attention was paid to studying the impact of NH_4_OH treatment on the intraparticle pore structure of the geopolymer *via* N_2_ physisorption combined with ^129^Xe and ^1^H relaxometry NMR techniques.

## 2. Experimental methods

### 2.1. Sample preparation

An alkali activating solution was first prepared by mixing 37.4 g sodium silicate solution (27% SiO_2_, 8% Na_2_O and 65% H_2_O) (VWR, BDH, Frankenwald, South Africa) with 5.6 g NaOH pellets (VWR, BDH, Frankenwald, South Africa). The mixing procedure was performed using a shear mixer (IKA, T 25 digital ULTRA-TURRAX, Staufen, Germany) at a speed of 3000 rpm for 5 min. Then, 31.55 g metakaolin (MetaMax, BASF, Ludwigshafen, Germany) was dissolved in the alkali-activating solution, with 3.85 mL H_2_O. The mixture was placed into silicone molds and sealed into a plastic bag for curing at room temperature. After 24 h of curing, the solid samples were ground into powders using a vibratory disc mill (Retsch RS200, RETSCH GmbH, Haan, Germany). The oxide molar ratio of this geopolymer was: Na_2_O : Al_2_O_3_ : SiO_2_ : H_2_O = 1.0 : 1.1 : 3.8 : 13.6.

Before the NH_4_OH treatment, a low-concentration acetic acid treatment was performed. 12.5 g of the geopolymer powder was added to 100 mL of 0.1 M acetic acid (VWR, Merck, Radnor, United States) and shaken on an orbital shaker (Advanced d3500 Orbital Shaker, VWR, Radnor, United States) for 10 min. The powders were then filtered out, while the pH of the filtered liquid was measured (Accumet model 20, Fisher Scientific, Vantaa, Finland). The geopolymer powders were then shaken with 0.1 M acetic acid and filtered repeatedly until the pH value of the filtered liquid became 7 to 8.

The four sets of 12.5 g of geopolymer powders were subjected to 500 mL of 0.02 M NH_4_OH (Sigma-Aldrich, Munich, Germany) solution treatment in a beaker, and stirred using a magnetic stirrer (Mixdrive 6, 2mag AG, Muenchen, Germany) at 600 rpm for 0 min, 15 min, 3 h and 24 h, and these four geopolymers treated samples were named N1, N2, N3 and N4, respectively. Before characterizing the four samples, they were dried overnight at 100 °C.

**Fig. 1:**
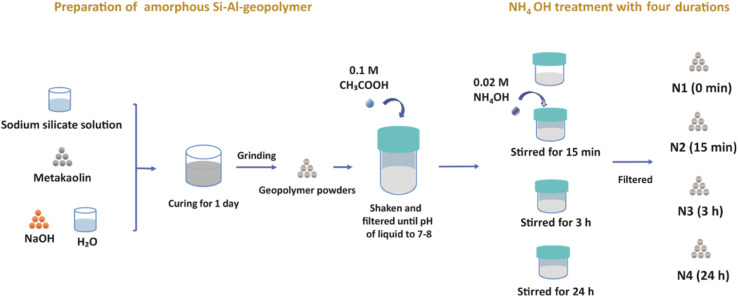
The procedure for sample preparation.

Here we would like to note that we used the low-concentration (0.02 M) NH_4_OH solution in order to avoid damaging the pore structure, which is expected to happen at higher concentrations. However, the 0.02 M concentration was too low to replace efficiently Na^+^ ions with NH_4_^+^ ions and thus it was hard to observe the effect of the NH_4_^+^ treatment. Therefore, an acetic acid treatment was applied before the NH_4_^+^ treatment in order to help exchange some original Na^+^ ions in geopolymer by H^+^ ions, thus facilitating the introduction of NH_4_^+^ ions into the pores (Fig. 1).

### 2.2. DRIFT spectra

DRIFT spectra in the range of 500–4000 cm^−1^ were collected for powder samples by a Bruker Vertex v80 spectrometer (Bruker, Rheinstetten, Germany).

### 2.3. X-ray fluorescence

X-ray fluorescence (XRF) measurements were used to determine the chemical composition of the samples. Scans were performed on an XRF spectrometer, PANalytical AXIOSmAX fitted with an Rh X-ray tube, which has a maximum power of 4 kW (Malvern PANanlytical, Malvern, UK).

### 2.4. Solid-state magic angle spinning (MAS) NMR

^27^Al and ^29^Si MAS NMR spectra were measured using a Bruker Avance III 7.05 T spectrometer (Bruker, Billerica, MA, USA). Samples were packed into 7 mm zirconia rotors and a 7 kHz spinning frequency was applied. For collecting ^27^Al MAS spectra, a single pulse sequence was used. The length of the excitation pulse was 1 μs. The number of scans was 1024 with a relaxation delay of 2 s. The experiment time was about 0.5 h. The ^27^Al spectra were collected on all four samples and referenced to the external reference of Al(NO_3_)_3_ at 0 ppm. The ^29^Si MAS spectra were also collected with the single pulse sequence. The pulse length was 4 μs. The spectra were accumulated for 8192 scans. The relaxation delay was 3 s and the experiment time was about 7 h. The ^29^Si spectra were referenced to tetramethylsilane (TMS) at 0 ppm.

### 2.5. Particle size

Particle size distributions were measured when geopolymer samples were dispersed into an NH_4_OH treatment solution by using a laser diffraction particle size analyser (LS 13320, Beckman Coulter, Inc., Brea, CA, USA). The induced optical model was ‘Fraunhofer. rfd’.

### 2.6. SEM images

SEM images were recorded using a Zeiss Ultra Plus field emission scanning electron microscope (FESEM) (Zeiss, Jena, Germany). The powdered geopolymers were carbon-coated before scanning.

### 2.7. N_2_ sorption

N_2_ sorption measurements were performed at −196 °C using Micromeritics ASAP 2020 (Micromeritics, Norcross, US). The four samples were degassed under vacuum at 100 °C, overnight. The pore size distributions of the mesopores were obtained using the Barret–Joyner–Halenda (BJH) method^33^ and the pore volumes of micropores were obtained using the *t*-plot method.^34^ The surface areas were analysed by Brunauer–Emmett–Teller (BET) method.^35^

### 2.8. ^129^Xe NMR

To control the particle size effect on the ^129^Xe NMR results, the samples were sieved with an air jet sieve (HOSOKAWA ALPINE 200LS, Hosokawa Alpine AG, Augsburg, Germany) before the experiments. The particles in the range of 90–250 μm were selected. However, this is not the actual particle size as this sieve cannot separate the aggregated small particles. The actual particle size distributions were measured while dispersed into iso-propanol using a laser diffraction particle size analyser (see Section 2.4).

The sample powders were loaded into a 5 mm NMR tube and 5–6 atm of the ^129^Xe gas was then condensed into each sample using liquid nitrogen and a vacuum system. After 5–6 atm of ^129^Xe gas was condensed into each sample using liquid nitrogen and a vacuum system, ^129^Xe NMR spectra were collected on Bruker Avance III 7.1 T spectrometer (Bruker, Billerica, MA, USA) with a 10 mm BBO probe. The ^129^Xe dynamic experiments, including measurements of the exchange rates and relaxation times (*T*_2_ and *T*_1_), were performed for samples N1, N2 and N3 on a Bruker Avance III 14.1 T spectrometer (Bruker, Billerica, MA, USA) with a 5 mm BBO probe.

^129^Xe NMR spectra were measured using a single pulse sequence. The data was collected using 64 scans and a 60 s relaxation delay between each scan. The experiment time of one ^129^Xe spectrum was about 1 hour. The variable temperature ^129^Xe spectra were collected for 11 temperature points in the range of 212–324 K. For 1 K of temperature increase, the sample was left for 5 min for temperature stabilization.

The exchange rates (*k*) were measured by ^129^Xe selective inversion recovery (IR) experiments^24^ at 298 K.^5^ The recovery time was varied from 10 μs to 1 s by 36 log-spaced steps. The length of the selective sinc pulse was 436 μs with 7 W power and the pulse was centred on the peak at 42–53 ppm (labelled with IP in Fig. 6). The number of scans was 128 and the relaxation delay was 10 s. The experiment time was about 15 h.

*T*_1_ of ^129^Xe was measured by the IR pulse sequence^36^ at 298 K. The recovery time increased from 100 μs to 1 s with 25 log-spaced steps. The number of scans was 64 and the relaxation delay was 10 s. The experiment time was about 6 h.

*T*_2_ of ^129^Xe was measured by the CPMG pulse sequence^37^ at 298 K. The echo time (2*τ*) was 160 μs. The number of echoes varied from 2 to 50 with 14 log-spaced steps. The number of scans was 256 and the relaxation delay was 10 s. The experiment time was about 10 h.

The *k*, *T*_1_ and *T*_2_ data fits are shown in Section S2.†

### 2.9. ^1^H NMR experiments

Before the ^1^H NMR experiments, the geopolymer samples were water-saturated for 14 days. ^1^H *T*_2_ and *T*_1_ measurements, as well as 2D ^1^H *T*_2_–*T*_2_ and *T*_1_–*T*_2_ experiments, were performed on a Magritek Spinsolve 43 MHz NMR spectrometer (Magritek, Aachen, Germany) at 298 K.

The spectrally resolved CPMG experiments were conducted to acquire *T*_2_ distributions. The echo time was 150 μs and 1000 echoes were collected in a single scan. The number of scans was 4 and the relaxation delay was 14 s. The experiment time was about 16 h.

The *T*_1_ distributions were acquired with the saturation recovery (SR) pulse sequence.^38^ The recovery time was varied from 1 ms to 2 s with 64 log-spaced steps. The number of scans was 4 and the repetition time was 14 s. The experiment time was about 1 h.

*T*_2_–*T*_2_ experiments^26^ were performed with an echo time equal to 150 μs and 1000 echoes were acquired in the direct dimension. In the indirect dimension, the echo number was varied from 2 to 2000 in 64 log-spaced steps. The number of scans was 128 and the relaxation delay was 6 s. The experiment time was about 14 h. The experiments were repeated with four different mixing times of 0.01, 0.02, 0.2 and 1 ms.

The *T*_1_ SR–*T*_2_ pulse sequence^26,27^ was used for collecting *T*_1_–*T*_2_ data. The echo time was 150 μs and 1000 echoes were collected in a single scan. The recovery time was varied from 1 ms to 5 s with 64 log-spaced steps. The number of scans was 128 and the relaxation delay was 6 s. The experiment time was about 14 h.

The ^1^H NMR cryoporometry experiments^30^ were performed on a Bruker Avance III 11.7 T spectrometer (Bruker, Billerica, MA, USA) with a 10 mm BBO probe. The cryoporometry experiments contained 56 variable temperature CPMG experiments over a temperature range of 170 to 276 K. A temperature stabilization delay of 5 min K^−1^ was used between experiments. The CPMG echo time was 200 μs and the number of echoes was 1000. The relaxation delay was 5 s and the number of scans was 64. The total experiment time for each sample was about 16 h. The data fits are shown in Section S3.†

1D *T*_2_ and *T*_1_ as well as 2D *T*_2_–*T*_2_ and *T*_1_–*T*_2_ distributions were obtained using the Laplace inversion implemented in MATLAB R2017b (Mathworks, Natick, Massachusetts, United States of America), provided by the research group of late Prof. P. Callaghan.^30,39,40^

## 3. Results and discussion

### 3.1. Ion-exchange and structure

**Fig. 2:**
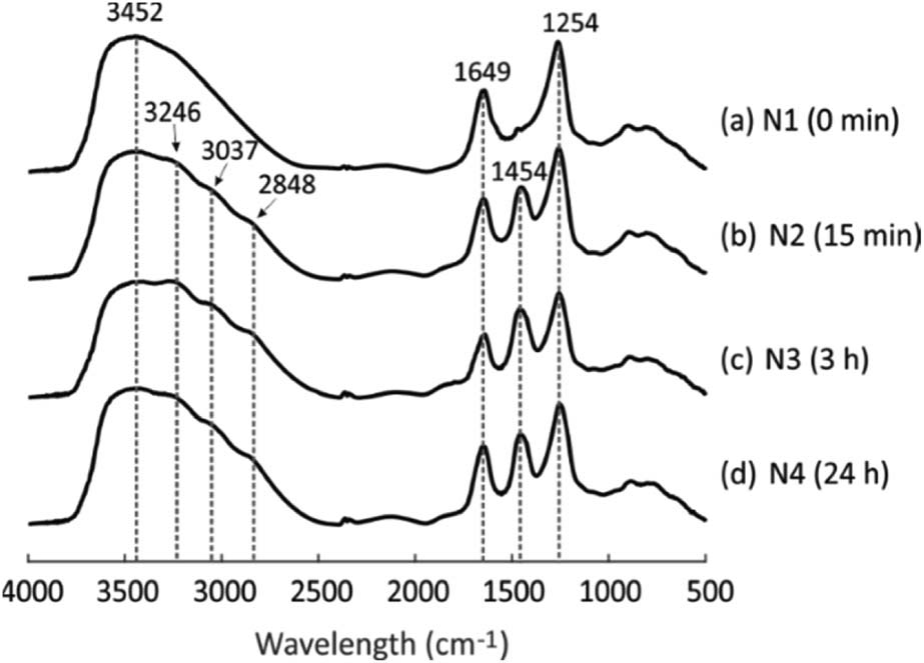
DRIFT spectra of four geopolymers after different NH_4_OH treatment times: (a) N1, (b) N2, (c) N3 and (d) N4.

DRIFT spectra were collected to detect the changes in the chemical structure of the geopolymer due to the NH_4_OH treatment. As visible in Fig. 2a, the geopolymer without the NH_4_OH treatment shows three main peaks at 3452, 1649 and 1254 cm^−1^, which are assigned to stretching H–O, bending H–O, and asymmetric stretching Si–O–T groups (T is Si or Al) in the geopolymer framework, respectively.^41,42^ After the geopolymer was treated with NH_4_OH for 15 min, 3 h and 24 h, three peaks appeared at 3246, 3037 and 2848 cm^−1^ (Fig. 2b–d), representing the appearance of anti-symmetric N–H stretching bands, as well as another new peak at 1454 cm^−1^, which is ascribed to the anti-symmetric bending H–N–H.^43,44^ These peaks provide evidence that NH_4_^+^ went into the pores after the NH_4_OH treatment and the NH_4_^+^-geopolymer was formed. This corresponds to the XRF measurement results: the weight percentage of Na_2_O decreased from 6.3 to 5.1 (Table 1), which is because Na^+^ was continuously exchanged with NH_4_^+^ during NH_4_OH treatment, although acetic acid (0.1 M) resulted in a larger level of exchange with a decrease from 11.6 to 6.3. The NH_4_^+^-exchanged geopolymer enabled the preparation of material with high acidity after calcination at temperature of 550–600 °C.^14,17^

**Table 1:** Weight percentage of Na_2_O and SiO_2_/Al_2_O_3_ molar ratio of the geopolymer before acetic acid treatment and four geopolymers (N1, N2, N3 and N4) after acetic acid and NH_4_OH treatment measured through XRF analysis

	Na_2_O (%)	SiO_2_/Al_2_O_3_ molar ratio
Geopolymer before acetic acid treatment	11.6	3.54
N1	6.26	3.13
N2	5.61	3.16
N3	4.14	3.14
N4	5.13	3.12

**Fig. 3:**
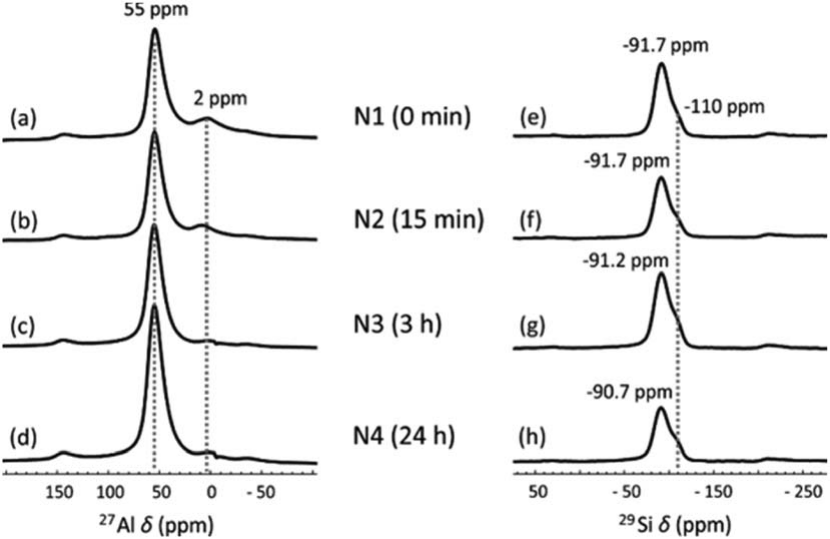
(a–d) ^27^Al and (e–h) ^29^Si MAS NMR spectra of (a and e) N1, (b and f) N2, (c and g) N3 and (d and h) N4.

The local Al and Si chemical environment changes after the NH_4_OH treatment were also characterized using ^27^Al and ^29^Si MAS NMR. In the ^27^Al NMR spectra (Fig. 3a–d), the peak at 55 ppm is assigned to Al in tetrahedral coordination and the small peak at 2 ppm to six-fold coordinated Al, arising from a small amount of non-framework Al.^45^ The treatment does not change the positions of the peaks, but the peak at 2 ppm gradually vanishes as NH_4_OH treatment time increases to 24 h. This may be because the extra-framework-Al was removed *via* the NH_4_OH treatment. A similar result was also found in zeolites.^32^ In ^29^Si MAS NMR spectra (Fig. 3e–h), two partially overlapping peaks are observed. The smaller peak at −110 ppm corresponds to SiQ_4_(0Al), which mainly results from unreacted metakaolin.^46^ The other peak is a typical broad geopolymer peak at −90 to −92 ppm. Normally, this peak contains four species, SiQ_4_(4Al), SiQ_4_(3Al), SiQ_4_(2Al) and SiQ_4_(1Al), arranged from low to high frequency.^47^ This peak shifts from −91.7 ppm (Fig. 3e) to −90.7 ppm (Fig. 3h) with increasing NH_4_OH treatment time. This small shift was also found for zeolite Y after the NH_4_OH treatment in a previous study, which is ascribed to the desilication impact of NH_4_OH.^32^ Removal of Si from the framework leads to an increased chemical shift. The decreasing input Si/Al ratio in the geopolymer framework was also previously found to lead to increasing chemical shifts.^47^ The desilication was also found from XRF measurements, where the SiO_2_-to-Al_2_O_3_ molar ratio was decreased from 3.16 to 3.12 as the NH_4_OH treatment time increased from 15 min to 24 h (Table 1).

### 3.2. Particle size

**Fig. 4:**
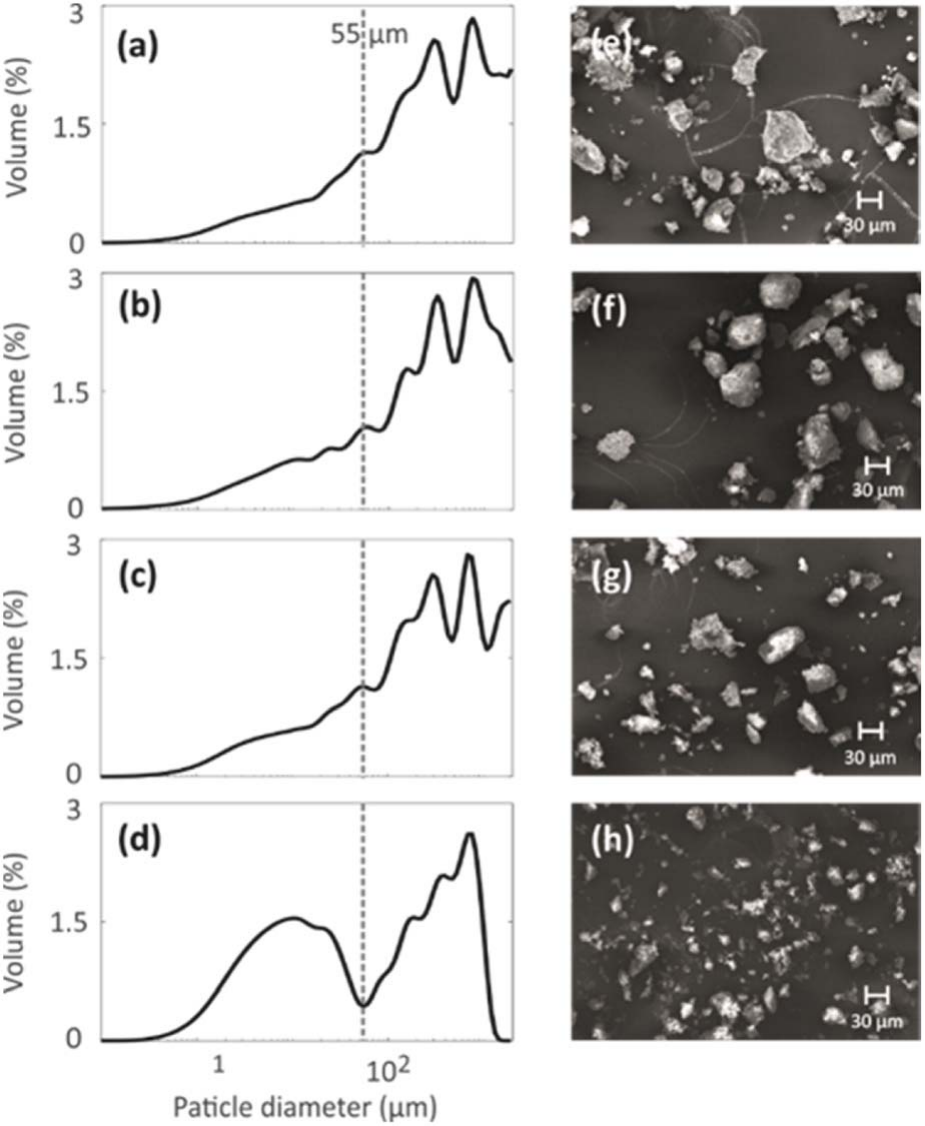
(a–d) Particle size distributions and (e–h) SEM images of four geopolymers with different NH_4_OH post-treatment times: (a and e) 0 min (N1), (b and f) 15 min (N2), (c and g) 3 h (N3) and (d and h) 24 h (N4).

Particle size distributions of the geopolymer powders were measured to investigate the effect of the mild NH_4_OH treatment on geopolymer particles. The particle size distributions shown in Fig. 4a–d indicate that the NH_4_OH treatment lasting for 0–3 h (N1, N2 and N3) does not alter the particle size. However, the NH_4_OH treatment lasting 24 h (N4) leads to a decrease in the particle size. The particles with similar sizes are visible in the SEM images (Fig. 4e–h). The reason for the decreased particle size is hypothesized to be the desilication caused by the NH_4_OH treatment. As the Si–O bonds break, the large particles become smaller while breaking into multiple smaller pieces. The formation of the crystal fragments of ZSM-5 zeolite due to desilication was also observed in a previous study.^48^

### 3.3. Pore structure

The effect of NH_4_OH treatment on the pore structure of the geopolymer was studied by N_2_ physisorption, ^1^H cryoporometry, ^1^H NMR relaxometry and ^129^Xe NMR methods as described below.

#### 3.3.1 Pore volume, pore surface area and pore size

Pore volumes, the average pore sizes, and the surface areas of the four samples were measured by N_2_ sorption isotherms (Table 2). The micropore volume decreases and the mesopore volume increases as the NH_4_OH treatment time increases. The average mesopore size was also found to increase. This means that NH_4_OH treatment enlarges some micropores to mesopores and already existing mesopores to bigger mesopores. The average pore surface area acquired from both BET and *t*-plot analysis in the system decreases as a function of the treatment time. This, as expected, shows the impact of the desilication caused by NH_4_OH treatment on enlarging micropores to mesopores and perhaps introducing additional mesopores within the geopolymer framework.

**Table 2:** Pore volumes, pore sizes and surface areas of the four geopolymers measured using the N_2_ sorption method

	Pore volume (cm^3^ g^−1^)		Pore size (nm)	Surface area (m^2^ g^−1^)	
	Micropore (*t*-plot)	Mesopore (BJH)	Mesopore (BJH)	Average (BET)	Micropore (*t*-plot)
N1	0.00385	0.256	6.54	130	14.1
N2	0.00321	0.261	6.61	126	12.5
N3	0.00213	0.264	6.66	123	10.1
N4	0.00130	0.274	7.43	112	7.89

**Fig. 5:**
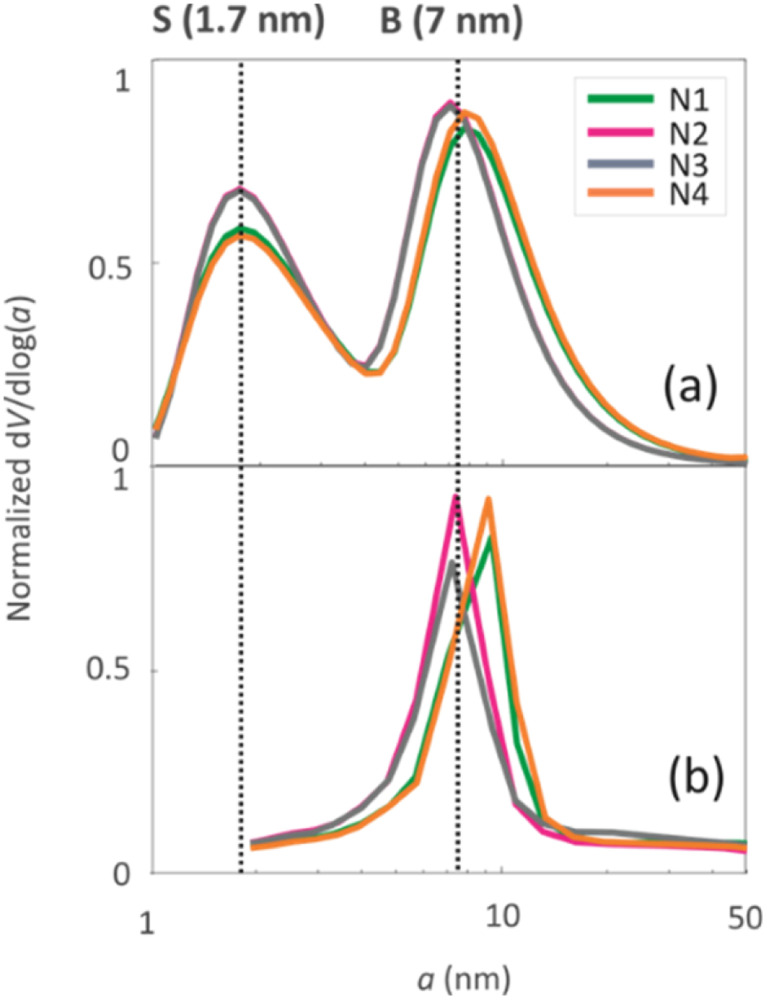
Pore size distributions of the four geopolymers derived from (a) ^1^H cryoporometry NMR data and (b) N_2_ desorption data analysed with the BJH method.

The pore size distributions of the samples were determined by N_2_ sorption^33^ and ^1^H NMR cryoporometry^30,31^ measurements. The N_2_ pore size distribution obtained by the BJH method (desorption curve) is limited to the mesoporous range, while the NMR cryoporometry can probe both micro- and mesopores.^49^ The pore size distributions (Fig. 5a) fitted from NMR cryoporometry (Fig. S3†) include two peaks for all the samples, one from micropores and another from mesopores. The mesopore peak is centred around 7 nm, being in good agreement with the single peak observed by N_2_ sorption (Fig. 5b). The micropore peak is centred around 1.7 nm. According to the N_2_ sorption data shown in Table 2, the micropore volume decreases while mesopore volume increases with longer NH_4_OH treatment time.

#### 3.3.2 Pore accessibility and exchange detected by ^129^Xe NMR

The pore accessibility was probed by ^129^Xe NMR, as the ^129^Xe chemical shift is able to distinguish intra and interparticle pores.^13^^129^Xe in a small pore has a larger chemical shift than ^129^Xe outside the pores.^22^

**Fig. 6:**
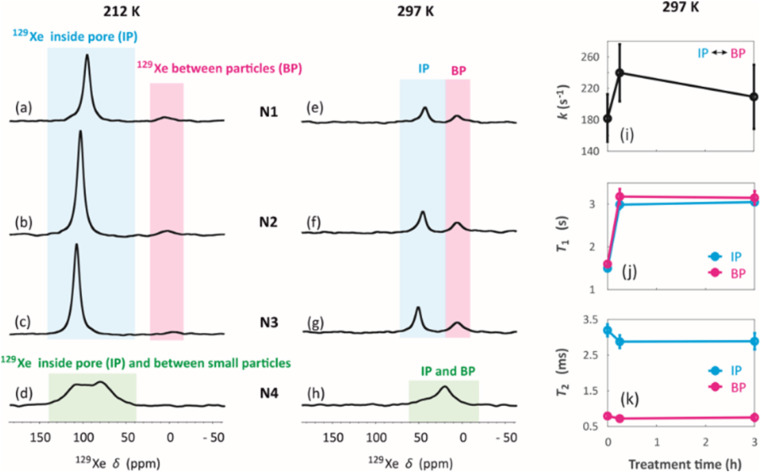
^129^Xe NMR spectra of (a and e) N1, (b and f) N2, (c and g) N3 and (d and h) N4 measured at (a–d) 212 K and (e–h) 297 K. (i) Exchange rates of Xe between the intra particle and inter particle sites in samples N1, N2 and N3 determined using selective inversion recovery experiments. (j) *T*_1_ and (k) *T*_2_ relaxation times of ^129^Xe in N1, N2 and N3 samples.

^129^Xe spectra were collected at variable temperatures from 212 to 324 K to study pore accessibility (Fig. S1†). As seen in Fig. 6, two peaks are observed for samples N1, N2 and N3 at low (212 K) and high (297 K) temperatures. The tall peak labelled as IP around 110 ppm at 212 K is assigned to Xe in small pores inside the particles. The chemical shift and its relatively small temperature dependency imply that the IP signal arises predominantly from Xe atoms in micropores.^5,22^ The small peak labelled as BP around 6 ppm is attributed to Xe between larger particles (55 to 250 μm).^5,22^ Due to the large interparticle void spaces, the chemical shift of the BP peak is close to the free gas shift and almost independent of temperature. In contrast, a single, very broad peak is observed for the N4 sample in the chemical shift range of 50 and 130 ppm at 212 K. This is interpreted to be a consequence of the smaller particle size of the N4 sample. According to Fig. 4d, contrary to other samples, the N4 sample has several particles in the range of 0.04 to 55 μm. Due to the decreased particle size, the exchange is faster between the intraparticle and interparticle pool, resulting in partial exchange average signals (intermediate exchange region).

Exchange rates *k* of Xe between the intraparticle and interparticle pores of N1, N2 and N3 were determined by selective inversion recovery experiments (Fig. S2†).^24^ The observed exchange rates (Fig. 6i) are in the range of 140–270 s^−1^, and within the error bars they are independent of the NH_4_OH treatment time.

^129^Xe *T*_1_ and *T*_2_ relaxation times (Fig. 6j and k) also reflect the dynamics of Xe in the geopolymer samples. Within the error bars, *T*_1_ relaxation times (1.5–3 s) of the IP and PB peaks are equal due to exchange averaging, as the relaxation rates (0.3–0.7 s^−1^) are much smaller than the exchange rates (140 to 270 s^−1^). *T*_1_ is longer (3 s) for the NH_4_OH-treated samples N2 and N3 than for the untreated N1 sample (1.5 s). This may be a consequence of changed surface interactions due to the partial Na^+^–NH_4_^+^ ion exchanges. *T*_2_ relaxation times (0.5–3.5 ms) are much shorter than the *T*_1_ relaxation times. According to relaxation modelling, fluctuations in the isotropic chemical shift are known to be dominating the *T*_2_ relaxation mechanism of ^129^Xe in porous materials because of the large chemical shift between the exchanging sites.^50^ Because *T*_2_ rates (300–2000 s^−1^) are higher than the exchange rates (140–270 s^−1^), *T*_2_ values are not exchange averaged; *T*_2_ of ^129^Xe in the intraparticle site is longer (about 3 ms) than that in the inter particle site (about 0.7 ms).

#### 3.3.3 Pore types and connectivity detected by ^1^H NMR relaxation

The 1D ^1^H *T*_2_ and *T*_1_, as well as 2D *T*_2_–*T*_2_ and *T*_1_–*T*_2_ measurements of the water absorbed in the geopolymer samples were performed to investigate how the pore types and connectivity change with the NH_4_OH treatment. When water is inside the geopolymer, *T*_2_ and *T*_1_ relaxation times reflect the pore size.^4,17^

**Fig. 7:**
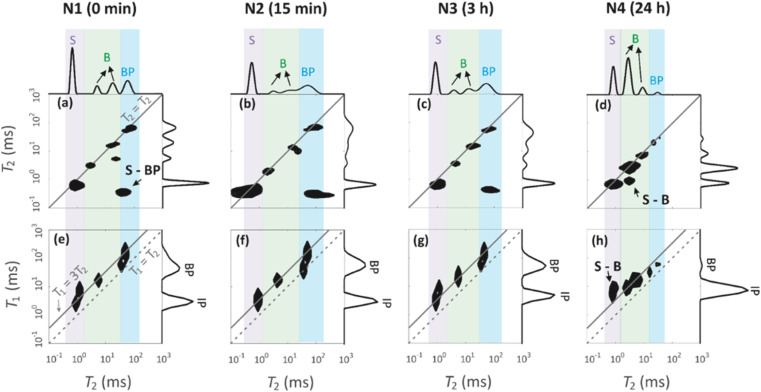
(a–d) ^1^H *T*_2_–*T*_2_ exchange spectra of water in the geopolymer samples measured with mixing time of 0.2 ms. (e–h) ^1^H *T*_1_–*T*_2_ correlation spectra. *T*_2_ and *T*_1_ distributions measured using CPMG and saturation recovery experiments are shown on the top and right of the 2D plots.

Four peaks are observed in the *T*_2_ distributions shown on the top of *T*_2_–*T*_2_ maps in Fig. 7a–d. The shortest *T*_2_ peak (label S) around *T*_2_ = 1 ms is interpreted to arise from water in the 1.7 nm micropores. The peaks (label B) in the region of 6–36 ms are assumed to represent water in the 7 nm mesopores. The longest *T*_2_ peak (label BP) is assigned for water in large voids in between the particles. The BP signal of the N4 sample has a smaller amplitude and shorter *T*_2_ than the other samples. This is because the smaller particles of N4 leave less space for the free water between the particles.

*T*_1_ distributions are shown on the right of the *T*_1_–*T*_2_ maps in Fig. 7e–h. *T*_1_ values are systematically longer than *T*_2_ values. The *T*_1_ distributions include two peaks around 5 and 50 ms, which are attributed to the water inside the intraparticle pores (IP) and the water inside the interparticle pores (BP). The micropore and mesopore peaks S and B observed in the *T*_2_ distributions are expected to fuse in the *T*_1_ distributions due to exchange; this implies that the micropore–mesopore exchange rate of water is between the *T*_1_ and *T*_2_ relaxation rates, *i.e.*, in the range of 200–1000 s^−1^. The cross-peak S-BP visible in the *T*_2_–*T*_2_ exchange maps (Fig. 7a–d) shows that there is also an exchange between the intraparticle and interparticle pools. The *T*_1_–*T*_2_ maps show the correlations between the *T*_1_ and *T*_2_ relaxation time values and confirm the above-mentioned interrelated assignments of the peaks in the *T*_1_ and *T*_2_ distributions.

## 4. Conclusions

We studied the effect of mild NH_4_OH treatment on a metakaolin-based geopolymer in terms of ion exchange, chemical structure, particle size, and pore structure.

(1) The obtained DRIFT, accompanied by XRF results shows that ion exchange of the charge balancing alkali cations (*i.e.*, Na^+^) with NH_4_^+^ was successfully achieved by this treatment.

(2) The solid-state NMR spectra and XRF data proved that the NH_4_OH treatment removed the extra-framework-Al from the geopolymer structure and also led to the desilication of the geopolymer framework.

(3) The particle size reduction resulting from NH_4_OH treatment was found by both laser diffraction particle-size analysis and SEM.

(4) After the NH_4_OH treatment, the pore volume and pore size were enlarged, but the pore accessibility and pore connectivity were not altered, as revealed by N_2_ sorption isotherms, ^129^Xe and ^1^H NMR.

The changes in the particle size and pore characteristics are considered to be due to desilication caused by NH_4_OH treatment.

In this study, the NH_4_OH treatment was carried out under mild conditions (0.02 M at room temperature) to ensure maintaining the structural integrity of the geopolymers. This resulted in limited ion-exchange efficiency as well as limited impact on the pore characteristics of the prepared materials. This work provides a new approach to simplifying the synthesis of ion-exchanged geopolymers with modified porosity as low-cost functional materials for catalysis and adsorption applications. Further investigation on the impact of NH_4_OH treatment under harsher conditions (for instance, NH_4_^+^ concentration ≥0.1 M, and moderate temperature range (323–353 K)) would be of high interest.

## Conflicts of interest

There are no conflicts to declare.

## Supplementary Material

RA-014-D4RA03972F-s001

## References

[cit1] Xu H., Van Deventer J. S. J. (2000). The geopolymerisation of alumino-silicate minerals. Int. J. Miner. Process..

[cit2] Sazama P., Bortnovsky O., Dědeček J., Tvarůžková Z., Sobalík Z. (2011). Geopolymer based catalysts—New group of catalytic materials. Catal. Today.

[cit3] Davidovits J. , Properties of Geopolymer Cements, 1994

[cit4] Li J., Mailhiot S., Sreenivasan H., Kantola A. M., Illikainen M., Adesanya E., Kriskova L., Telkki V.-V., Kinnunen P. (2021). Curing process and pore structure of metakaolin-based geopolymers: Liquid-state 1H NMR investigation. Cem. Concr. Res..

[cit5] Li J., Mailhiot S., Sreenivasan H., Kantola A. M., Telkki V.-V., Kinnunen P. (2022). 129Xe NMR analysis reveals efficient gas transport between inborn micro-, meso- and macropores in geopolymers. Cem. Concr. Res..

[cit6] Liu Z., Ihl Woo S. (2006). Recent Advances in Catalytic DeNOX Science and Technology. Catal. Rev..

[cit7] Tang N., Yang K., Alrefaei Y., Dai J.-G., Wu L.-M., Wang Q. (2020). Reduce VOCs and PM emissions of warm-mix asphalt using geopolymer additives. Constr. Build. Mater..

[cit8] Tan T. H., Mo K. H., Ling T.-C., Lai S. H. (2020). Current development of geopolymer as alternative adsorbent for heavy metal removal. Environ. Technol. Innovation.

[cit9] Rasaki S. A., Bingxue Z., Guarecuco R., Thomas T., Minghui Y. (2019). Geopolymer for use in heavy metals adsorption, and advanced oxidative processes: A critical review. J. Cleaner Prod..

[cit10] Alzeer M. I. M., MacKenzie K. J. D., Keyzers R. A. (2017). Facile synthesis of new hierarchical aluminosilicate inorganic polymer solid acids and their catalytic performance in alkylation reactions. Microporous Mesoporous Mater..

[cit11] Chen H., Zhang Y. J., He P. Y., Li C. J., Liu L. C. (2020). Facile synthesis of cost-effective iron enhanced hetero-structure activated carbon/geopolymer composite catalyst for NH3-SCR: Insight into the role of iron species. Appl. Catal., A.

[cit12] Zhang Y. J., Liu L. C., Ni L. L., Wang B. L. (2013). A facile and low-cost synthesis of granulated blast furnace slag-based cementitious material coupled with Fe2O3 catalyst for treatment of dye wastewater. Appl. Catal., B.

[cit13] Alzeer M. I. M., MacKenzie K. J. D., Keyzers R. A. (2016). Porous aluminosilicate inorganic polymers (geopolymers): a new class of environmentally benign heterogeneous solid acid catalysts. Appl. Catal., A.

[cit14] Verboekend D., Vilé G., Pérez-Ramírez J. (2012). Hierarchical Y and USY Zeolites Designed by Post-Synthetic Strategies. Adv. Funct. Mater..

[cit15] Karakus S. , New Trends in Ion Exchange Studies, BoD – Books on Demand, 2018

[cit16] O'Connor S. J., MacKenzie K. J. D., Smith M. E., Hanna J. V. (2010). Ion exchange in the charge-balancing sites of aluminosilicate inorganic polymers. J. Mater. Chem..

[cit17] Barbosa T. R., Foletto E. L., Dotto G. L., Jahn S. L. (2018). Preparation of mesoporous geopolymer using metakaolin and rice husk ash as synthesis precursors and its use as potential adsorbent to remove organic dye from aqueous solutions. Ceram. Int..

[cit18] Wisser D., Hartmann M. (2021). 129Xe NMR on Porous Materials: Basic Principles and Recent Applications. Adv. Mater. Interfaces.

[cit19] Song Y.-Q. (2013). Magnetic Resonance of Porous Media (MRPM): A perspective. J. Magn. Reson..

[cit20] Javed M. A., Komulainen S., Daigle H., Zhang B., Vaara J., Zhou B., Telkki V.-V. (2019). Determination of pore structures and dynamics of fluids in hydrated cements and natural shales by various 1H and 129Xe NMR methods. Microporous Mesoporous Mater..

[cit21] Zhou B., Komulainen S., Vaara J., Telkki V.-V. (2017). Characterization of pore structures of hydrated cements and natural shales by 129Xe NMR spectroscopy. Microporous Mesoporous Mater..

[cit22] Terskikh V. V., Moudrakovski I. L., Breeze S. R., Lang S., Ratcliffe C. I., Ripmeester J. A., Sayari A. (2002). A General Correlation for the 129Xe NMR Chemical Shift−Pore Size Relationship in Porous Silica-Based Materials. Langmuir.

[cit23] Romanenko K. V., Fonseca A., Dumonteil S., Nagy J. B., d'Espinose de Lacaillerie J.-B., Lapina O. B., Fraissard J. (2005). 129Xe NMR study of Xe adsorption on multiwall carbon nanotubes. Solid State Nucl. Magn. Reson..

[cit24] Bain A. D., Cramer J. A. (1993). A Method for Optimizing the Study of Slow Chemical Exchange by NMR Spin-Relaxation Measurements. Application to Tripodal Carbonyl Rotation in a Metal Complex. J. Magn. Reson., Ser. A.

[cit25] Lee J. H., Labadie C., Springer C. S., Harbison G. S. (1993). Two-dimensional inverse Laplace transform NMR: altered relaxation times allow detection of exchange correlation. J. Am. Chem. Soc..

[cit26] Snaar J. E. M., Van As H. (1969). A method for the simultaneous measurement of NMR spin-lattice and spin-spin relaxation times in compartmentalized systems. J. Magn. Reson..

[cit27] Song Y.-Q., Venkataramanan L., Hürlimann M. D., Flaum M., Frulla P., Straley C. (2002). *T*1–*T*2 Correlation Spectra Obtained Using a Fast Two-Dimensional Laplace Inversion. J. Magn. Reson..

[cit28] Strange J. H., Rahman M., Smith E. G. (1993). Characterization of porous solids by NMR. Phys. Rev. Lett..

[cit29] Petrov O. V., Furó I. (2009). NMR cryoporometry: Principles, applications and potential. Prog. Nucl. Magn. Reson. Spectrosc..

[cit30] Valckenborg R. M. E., Pel L., Kopinga K. (2002). Combined NMR cryoporometry and relaxometry. J. Phys. D: Appl. Phys..

[cit31] Aksnes D. W., Førland K., Kimtys L. (2001). Pore size distribution in mesoporous materials as studied by 1 H NMR. Phys. Chem. Chem. Phys..

[cit32] Van Aelst J., Verboekend D., Philippaerts A., Nuttens N., Kurttepeli M., Gobechiya E., Haouas M., Sree S. P., Denayer J. F. M., Martens J. A., Kirschhock C. E. A., Taulelle F., Bals S., Baron G. V., Jacobs P. A., Sels B. F. (2015). Catalyst Design by NH4OH Treatment of USY Zeolite. Adv. Funct. Mater..

[cit33] Barrett E. P., Joyner L. G., Halenda P. P. (1951). The Determination of Pore Volume and Area Distributions in Porous Substances. I. Computations from Nitrogen Isotherms. J. Am. Chem. Soc..

[cit34] Lippens B. C., de Boer J. H. (1965). Studies on pore systems in catalysts: V. The t method. J. Catal..

[cit35] Pickett G. (1945). Modification of the Brunauer—Emmett—Teller Theory of Multimolecular Adsorption. J. Am. Chem. Soc..

[cit36] Vold R. L., Waugh J. S., Klein M. P., Phelps D. E. (1968). Measurement of Spin Relaxation in Complex Systems. J. Chem. Phys..

[cit37] Meiboom S., Gill D. (1958). Modified Spin-Echo Method for Measuring Nuclear Relaxation Times. Rev. Sci. Instrum..

[cit38] Markley J. L., Horsley W. J., Klein M. P. (1971). Spin-Lattice Relaxation Measurements in Slowly Relaxing Complex Spectra. J. Chem. Phys..

[cit39] Godefroy S., Callaghan P. T. (2003). 2D relaxation/diffusion correlations in porous media. Magn. Reson. Imaging.

[cit40] Venkataramanan L., Song Y.-Q., Hurlimann M. D. (2002). Solving Fredholm integrals of the first kind with tensor product structure in 2 and 2.5 dimensions. IEEE Trans. Signal Process..

[cit41] Aredes F. G. M., Campos T. M. B., Machado J. P. B., Sakane K. K., Thim G. P., Brunelli D. D. (2015). Effect of cure temperature on the formation of metakaolinite-based geopolymer. Ceram. Int..

[cit42] Mohd Basri M. S., Mustapha F., Mazlan N., Ishak M. R. (2021). Rice Husk Ash-Based Geopolymer Binder: Compressive Strength, Optimize Composition, FTIR Spectroscopy, Microstructural, and Potential as Fire-Retardant Material. Polymers.

[cit43] Mookherjee M., Welch M. D., Pollès L. L., Redfern S. A. T., Harlov D. E. (2005). Ammonium ion behaviour in feldspar: variable-temperature infrared and 2H NMR studies of synthetic buddingtonite, N(D,H)4AlSi3O8. Phys. Chem. Miner..

[cit44] Mihailova I. , Uzunov I. and Mehandjiev D., Waste Copper Slag/Aluminium Dross-Based Geopolymer, 2021

[cit45] He P., Wang M., Fu S., Jia D., Yan S., Yuan J., Xu J., Wang P., Zhou Y. (2016). Effects of Si/Al ratio on the structure and properties of metakaolin based geopolymer. Ceram. Int..

[cit46] Wang M. R., Jia D. C., He P. G., Zhou Y. (2010). Influence of calcination temperature of kaolin on the structure and properties of final geopolymer. Mater. Lett..

[cit47] Wan Q., Rao F., Song S., García R. E., Estrella R. M., Patiño C. L., Zhang Y. (2017). Geopolymerization reaction, microstructure and simulation of metakaolin-based geopolymers at extended Si/Al ratios. Cem. Concr. Compos..

[cit48] Yoo W. C., Zhang X., Tsapatsis M., Stein A. (2012). Synthesis of mesoporous ZSM-5 zeolites through desilication and re-assembly processes. Microporous Mesoporous Mater..

[cit49] Mitchell J., Webber J. B. W., Strange J. H. (2008). Nuclear magnetic resonance cryoporometry. Phys. Rep..

[cit50] Håkansson P., Asadullah Javed M., Komulainen S., Chen L., Holden D., Hasell T., Cooper A., Lantto P., Telkki V.-V. (2019). NMR relaxation and modelling study of the dynamics of SF 6 and Xe in porous organic cages. Phys. Chem. Chem. Phys..

