# Supplementary material for: Impact of NH4OH treatment on the ion exchange and pore characteristics of a metakaolin-based geopolymer
Source: RSC Adv. 2024 Jun 20;14(28):19935–44. doi: 10.1039/d4ra03972f (PMC11187750; doi:10.1039/d4ra03972f)
Supplement: RA-014-D4RA03972F-s001 [file RA-014-D4RA03972F-s001.pdf]

Electronic Supplementary Material (ESI) for RSC Advances.
This journal is © The Royal Society of Chemistry 2024

Impact of NH_4OH treatment on the ion-exchange and pore characteristics of metakaolin-based geopolymer

Jing Li[†‡ⱶ]*, Sarah Mailhiot [‡], Mohammad I.M. Alzeer[†], Tero Luukkonen[†], Anu M. Kantola[‡], Ville-Veikko Telkki[‡], Paivo Kinnunen[†]

[†] Fibre and Particle Engineering Research Unit, Faculty of Technology, P.O.Box 4300, FIN-90014, University of Oulu, Oulu, Finland

[‡] NMR Research Unit, Faculty of Science, P.O.Box 3000, FIN-90014, University of Oulu, Oulu, Finland

[ⱶ] NIMBE, CEA, CNRS, Université de Paris Saclay, CEA Saclay, 91191 Gif-sur-Yvette, France

* Corresponding author. E-mail address: Jing2.li@cea.fr

Contents

1. Variable-temperature ^{129}Xe spectra and fits....2
2. ^{129}Xe dynamic data and fits....3
3. ^{1}H cryoporometry data and fits 6

1. Variable-temperature ^{129}Xe spectra and fits

^{129}Xe spectra collected under various temperatures of four samples are shown in Fig. S1.

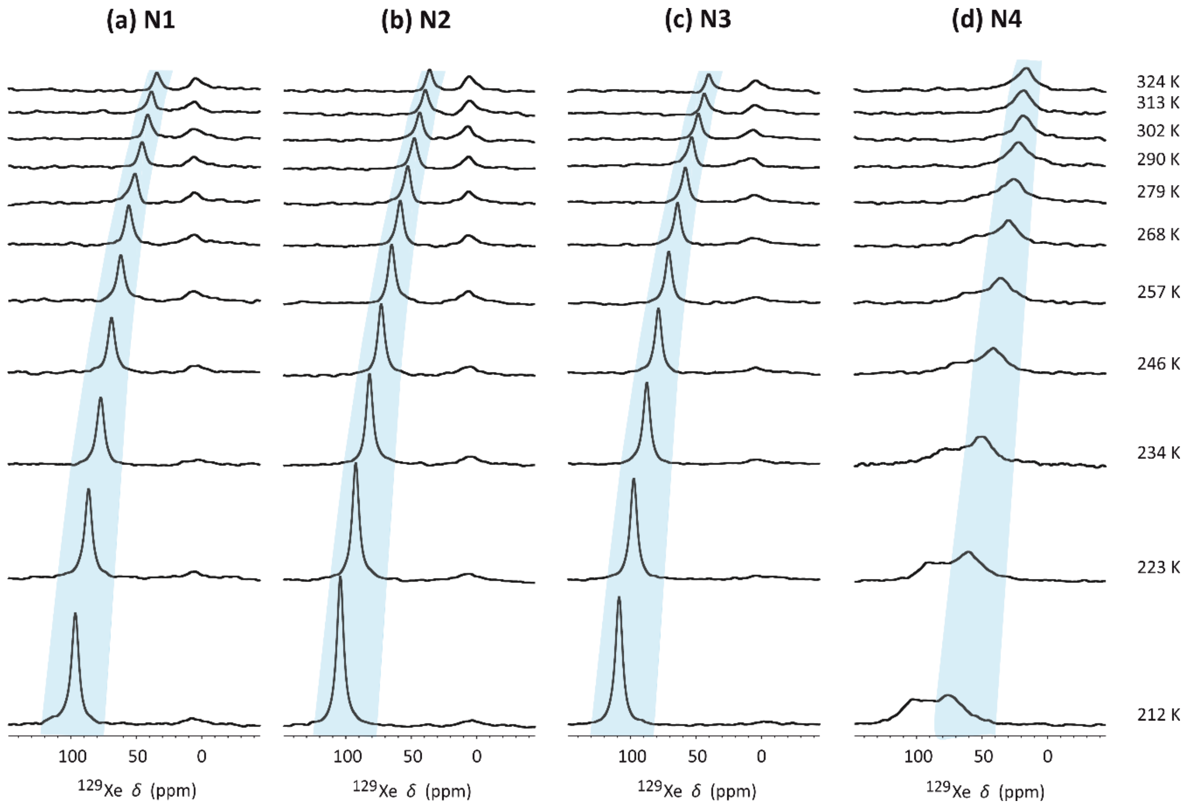

Figure S1. The ^{129}Xe spectra acquired under variable temperature of (a) N1, (b) N2, (c) N3 and (d) N4.

2. ^{129}Xe dynamic data and fits

The ^{129}Xe dynamic in site IP and BP were detected by measuring exchange rate (*k*), spin-lattice relaxation time (T_1) and spin-spin relaxation time (T_2). Selective-Inversion-Recovery (IR),[1] IR,[2] and Carr-Purcell-Meiboom-Gill (CPMG)[3] experiments were performed to get these three values, respectively. All of these data were analysed according to the following theories in MATLAB R2017b (Mathworks, Natick, Massachusetts, United States of America).

Based on the previous studies, [1,4,5] when the exchange process takes place between two sites, IP and BP, the time dependence of their magnetizations under ^{129}Xe selective-IR experiment is given by,

$$\frac{\partial}{\partial t}\begin{pmatrix} M_{IP}(t)-M_{eq,IP} \\ M_{BP}(t)-M_{eq,BP} \end{pmatrix} = -\begin{pmatrix} R_{1,IP}+k_{IP-BP} & -\frac{M_{\mathrm{eq,IP}}}{M_{\mathrm{eq,BP}}}*k_{\mathrm{IP-BP}} \\ -k_{IP-BP} & R_{1,BP}+\frac{M_{\mathrm{eq,IP}}}{M_{\mathrm{eq,BP}}}*k_{\mathrm{IP-BP}} \end{pmatrix}\begin{pmatrix} M_{IP}(t)-M_{eq,IP} \\ M_{BP}(t)-M_{eq,BP} \end{pmatrix}, \quad (S1)$$

where $M_{\mathrm{IP}}(t)$ and $M_{\mathrm{BP}}(t)$ are the magnetizations for two sites at time *t*, $M_{\mathrm{eq,IP}}$ and $M_{\mathrm{eq,BP}}$ are the equilibrium magnetizations, $R_{1,\mathrm{IP}}$ and $R_{1,\mathrm{BP}}$ are the spin-lattice relaxation rates ($1/T_1$) for two sites, $k_{\mathrm{IP\text{-}BP}}$ is the exchange rate between two sites.

Then our ^{129}Xe selective-IR data were fitted using the solution of Eq. S1, which is,

$$\begin{pmatrix} M_{IP}(t) \\ M_{BP}(t) \end{pmatrix} = \begin{pmatrix} M_{eq,IP} \\ M_{eq,BP} \end{pmatrix} + exp\left[-\begin{pmatrix} R_{1,IP}+k_{IP-BP} & -\frac{M_{\mathrm{eq,IP}}}{M_{\mathrm{eq,BP}}}*k_{\mathrm{IP-BP}} \\ -k_{IP-BP} & R_{1,BP}+\frac{M_{\mathrm{eq,IP}}}{M_{\mathrm{eq,BP}}}*k_{\mathrm{IP-BP}} \end{pmatrix} t\right]\begin{pmatrix} M_{IP}(0)-M_{eq,IP} \\ M_{BP}(0)-M_{eq,BP} \end{pmatrix}, \quad (S2)$$

ere $M_{\mathrm{IP}}(0)$ and $M_{\mathrm{BP}}(0)$ are the magnetizations at *t*=0.

The ^{129}Xe T_1IR signals intensities of peak IP and BP were derived by Topspin software (Bruker, Rheinstetten, Germany) and then fitted by the following equation, respectively,

$$M(t) = M_{eq} * \left[1 - n * \exp\left(\frac{-t}{T_1}\right)\right], \quad (S3)$$

where $M(t)$ is the magnetization at time *t*, M_{eq} are the magnetization at equilibrium-state, T_1 is the spin-lattice relaxation time and *n* is a free fitting parameter.

The fit equation for ^{129}Xe CPMG data is,

$$M(t) = M_{eq} * \exp\left(\frac{-t}{T_2}\right), \quad (S4)$$

where $M(t)$ is the magnetization at time *t*, M_{eq} are the magnetization at equilibrium-state, T_2 is the spin-spin relaxation time.

The raw signals and fitted signals of three experiments are shown in Fig. S2. The detailed fit results of these signals are shown in Tables S1-3.

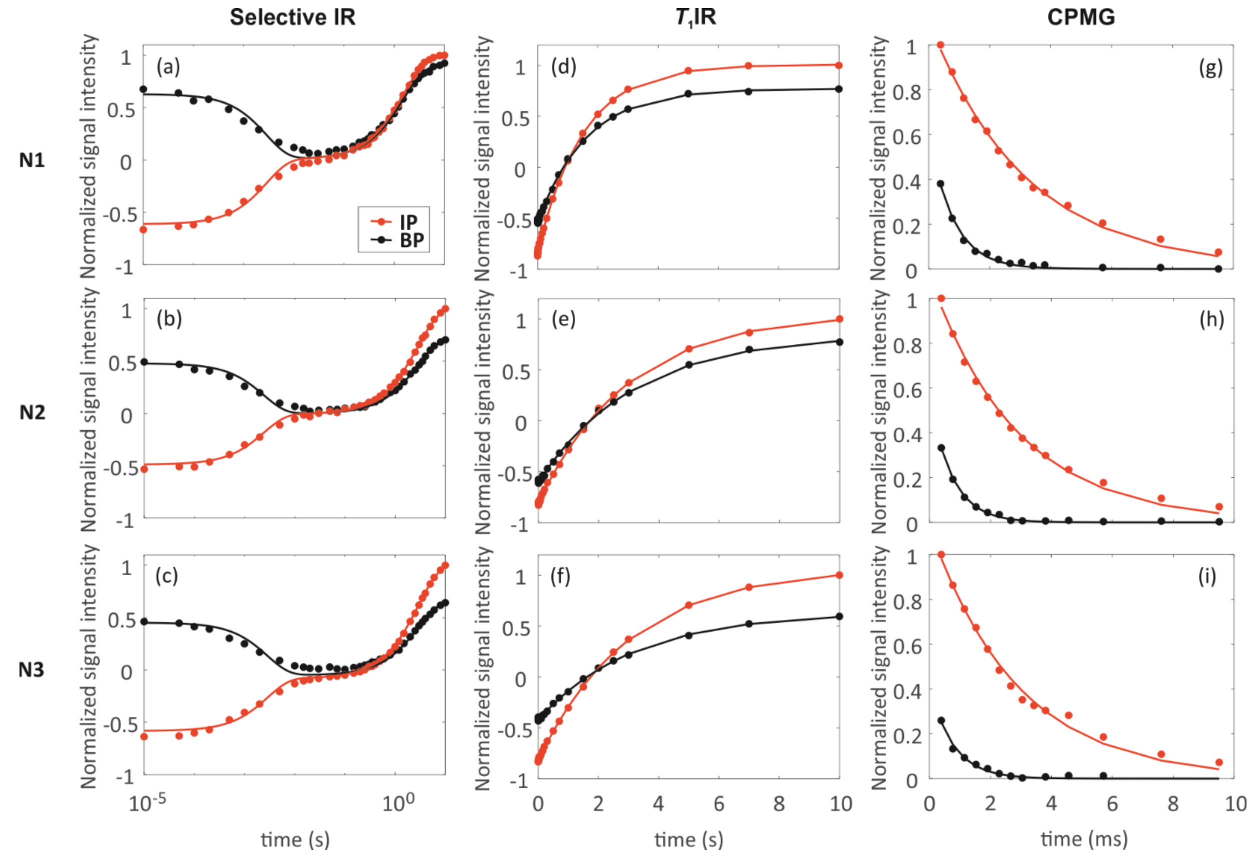

Figure S2. The raw signal (dots) and fitted signal (lines) of (a-c) ^{129}Xe selective IR, (d-f) T_1IR and (g-i) CPMG experiments performed at room temperature of (a, d and g) N1, (b, e and h) N2 and (c, f and i) N3.

Table S1. The fit results of ^{129}Xe selective-IR data.

SelIR	M_{eq} (IP)	M_{eq} (BP)	R_1 (IP)	R_1 (BP)	$k_{IP\text{-}BP}$	M_0 (IP)	M_0 (BP)	resnorm
**N 1**	0.99 ± 0.03	0.91 ± 0.03	1.33e-3 ± 4.79	1.39 ± 5.25	**181.67 ± 30.81**	-0.61 ± 0.03	0.63 ± 0.03	0.075
**N 2**	1.01 ± 0.03	0.73 ± 0.03	4.41e-3 ± 3.48	0.85 ± 4.86	**239.77 ± 36.23**	-0.49 ± 0.02	0.48 ± 0.02	0.037
**N 3**	1.02 ± 0.05	0.67 ± 0.04	4.84e-3 ± 3.57	0.87 ± 5.46	**209.22 ± 40.75**	-0.58 ± 0.03	0.45 ± 0.03	0.066

Table S2. The fit results of ^{129}Xe T_1IR data.

T_1IR	**N 1**		**N 2**		**N 3**	
	**IP**	**BP**	**IP**	**BP**	**IP**	**BP**
M_{eq}	1.01 ± 0.02	0.77 ± 0.02	1.06 ± 0.03	0.85 ± 0.04	1.07 ± 0.02	0.63 ± 0.02
**T_1 (s)**	**1.50 ± 0.04**	**1.60 ± 0.07**	**2.99 ± 0.10**	**3.18 ± 0.18**	**3.05 ± 0.07**	**3.15 ± 0.16**
**n**	1.83 ± 0.02	1.70 ± 0.02	1.76 ± 0.02	1.71 ± 0.03	1.77 ± 0.02	1.67 ± 0.03
**resnorm**	0.004	0.005	0.003	0.006	0.002	0.002

Table S3. The fit results of ^{129}Xe CPMG data.

CPMG	**N 1**		**N 2**		**N 3**	
	**IP**	**BP**	**IP**	**BP**	**IP**	**BP**
M_{eq}	1.11 ± 0.04	0.61 ± 0.05	1.10 ± 0.04	0.57 ± 0.03	1.12 ± 0.06	0.42 ± 0.05
**T_2 (ms)**	**3.20 ± 0.17**	**0.79 ± 0.08**	**2.88 ± 0.18**	**0.72 ± 0.04**	**2.89 ± 0.23**	**0.75 ± 0.10**
**resnorm**	0.004	0.001	0.005	4e-4	0.009	0.001

3. [1]H cryoporometry data and fits

The signal intensity at each temperature step ($S(X)$) is dependent on the inverse temperature, X=1000/T. Their relationship agrees with the following equation,[6]

$$S(X) = \sum_{i=1}^{n} \frac{S_{0i}}{2}\left[1 - \mathrm{erf}\left\{\frac{X - X_{ci}}{\sqrt{2}\sigma_i}\right\}\right], \quad (S5)$$

where n is the number of phase transitions, S_{0i}, X_{ci} and σ_i are the signal intensity, the inverse transition temperature and the width of the temperature distribution curve of phase i, respectively.

Our signal intensity data as a function of X are shown in Fig. S3. The data were fitted with Eq. S5, assumed with containing three components (n=3), S, B and V, by using CFTOOL installed in MATLAB R2017b (Mathworks, Natick, Massachusetts, United States of America). The detailed fit results are shown in Tab. S5.

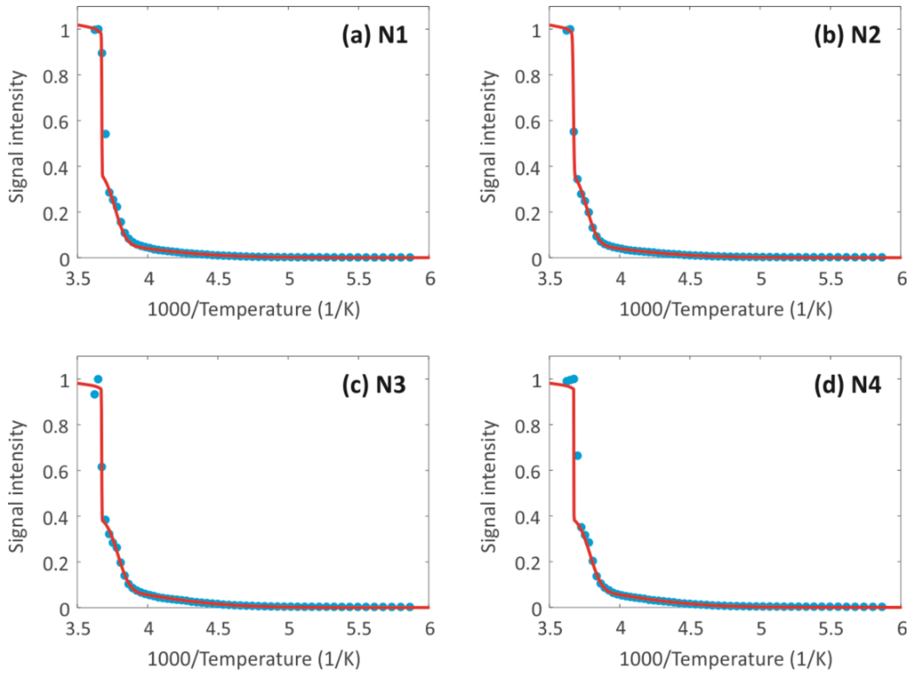

Figure S3. The raw (dots) signal intensity at each temperature step and the fitted (lines) signal intensity as a function of 1000/Temperature of (a) N1, (b) N2, (c) N3 and (d) N4.

Table S4. Fit results of cryoporometry data.

		$S_0/2$	X_c	$\sqrt{2}\sigma$	R^2
**N1**	**S**	0.15 ± 0.03	3.78 ± 0.01	0.09 ± 0.02	0.9997
	**B**	0.09 ± 0.25	3.50 ± 1.64	0.91 ± 0.73	
	**BP**	0.31 ± 0.02	3.67 ± 1e12	0.003 ± 3e12	
**N2**	**S**	0.15 ± 0.04	3.79 ± 0.01	0.09 ± 0.03	0.9983
	**B**	0.07 ± 0.17	3.88 ± 1.45	0.73 ± 0.83	
	**BP**	0.29 ± 0.01	3.67 ± 1e12	0.002 ± 7e12	
**N3**	**S**	0.15 ± 0.04	3.79 ± 0.02	0.09 ± 0.03	0.9983
	**B**	0.07 ± 0.16	3.89 ± 1.41	0.73 ± 0.81	
	**BP**	0.29 ± 0.02	3.67 ± 4e11	0.006 ± 2e12	
**N4**	**S**	0.15 ± 0.04	3.79 ± 0.01	0.09 ± 0.03	0.9997
	**B**	0.07 ± 0.16	3.89 ± 1.40	0.73 ± 0.81	
	**BP**	0.29 ± 0.02	3.67 ± 2e12	0.0005 ± 7e12	

By plugging the fitted $S_0/2$, X_c and $\sqrt{2}\sigma$ into the following equation, the pore size distribution dS/da as a function of a were obtained,

$$\frac{dS}{da} = \frac{10^3 k}{\sqrt{2\pi}\left(aT_{melting} - k\right)^2}\sum_{i=1}^{n}\frac{S_{0i}}{\sigma_i} \times \left[-\left\{\frac{10^3 a - X_{ci}\left(aT_{melting} - k\right)}{\sqrt{2}\sigma_i\left(aT_{melting} - k\right)}\right\}^2\right], \qquad (S6)$$

where $T_{melting}$ is the melting temperature of bulk water, which is 273 K in our cases, k is a constant, 89 K*nm is used here. The k has the same value as our pervious geopolymer sample[7] we used to evaluate the K_0 in Section S3, and it also agrees with the k value used for silica gel from one study.[8]

Reference

1 A. D. Bain and J. A. Cramer, A Method for Optimizing the Study of Slow Chemical Exchange by NMR Spin-Relaxation Measurements. Application to Tripodal Carbonyl Rotation in a Metal Complex, *Journal of Magnetic Resonance, Series A*, 1993, **103**, 217–222.

2 R. L. Vold, J. S. Waugh, M. P. Klein and D. E. Phelps, Measurement of Spin Relaxation in Complex Systems, *The Journal of Chemical Physics*, 1968, **48**, 3831–3832.

3 S. Meiboom and D. Gill, Modified Spin-Echo Method for Measuring Nuclear Relaxation Times, *Review of Scientific Instruments*, 1958, **29**, 688–691.

4 A. D. Bain and J. A. Cramer, Slow Chemical Exchange in an Eight-Coordinated Bicentered Ruthenium Complex Studied by One-Dimensional Methods. Data Fitting and Error Analysis, *Journal of Magnetic Resonance, Series A*, 1996, **118**, 21–27.

5 J. Li, S. Mailhiot, H. Sreenivasan, A. M. Kantola, V.-V. Telkki and P. Kinnunen, 129Xe NMR analysis reveals efficient gas transport between inborn micro-, meso- and macropores in geopolymers, *Cement and Concrete Research*, 2022, **155**, 106779.

6 D. W. Aksnes, K. Førland and L. Kimtys, Pore size distribution in mesoporous materials as studied by 1H NMR, *Phys. Chem. Chem. Phys.*, 2001, **3**, 3203–3207.

7 J. Li, S. Mailhiot, H. Sreenivasan, A. M. Kantola, M. Illikainen, E. Adesanya, L. Kriskova, V.-V. Telkki and P. Kinnunen, Curing process and pore structure of metakaolin-based geopolymers: Liquid-state 1H NMR investigation, *Cement and Concrete Research*, 2021, **143**, 106394.

8 R. M. E. Valckenborg, L. Pel and K. Kopinga, Combined NMR cryoporometry and relaxometry, *J. Phys. D: Appl. Phys.*, 2002, **35**, 249–256.
